# Supplementary material for: Predictable recovery rates in near-surface materials after earthquake damage
Source: Nat Commun. 2025 Feb 20;16:1790. doi: 10.1038/s41467-025-57151-8 (PMC11842714; doi:10.1038/s41467-025-57151-8)
Supplement: Supplementary file 1 — Supplementary Information [file 41467_2025_57151_MOESM1_ESM.pdf]

Predictable recovery rates in near-surface materials after earthquake damage
Supplementary information

Supplementary Figures and Table

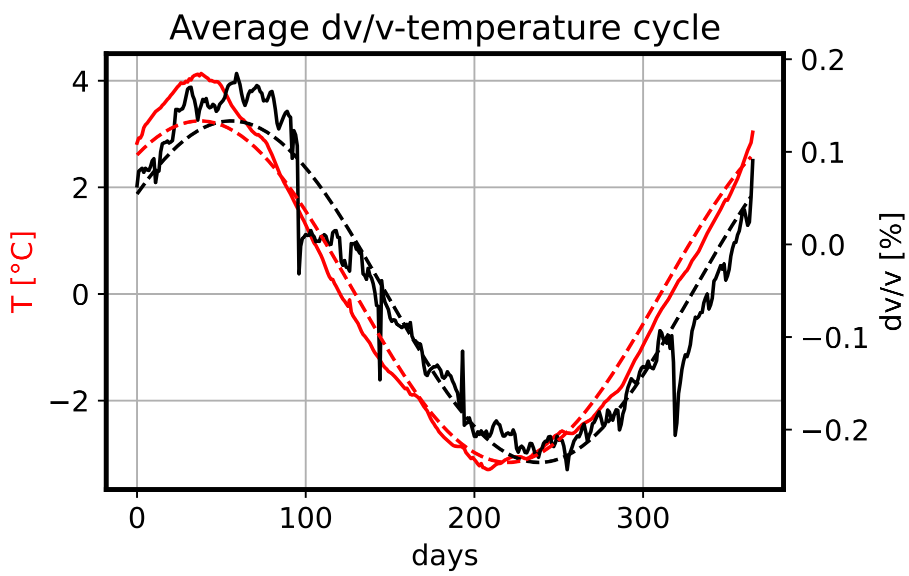

Figure S1. *Average annual cycle of surface temperature and dv/v variations in Patache, Chile.* The red solid curve shows the average relative temperature variation of the surface temperature time-series (shown in figure S2a). The solid black line shows the annual average for the dv/v variations. Dashed lines correspond to the sinusoidal fits of the data. The phase lag between the temperature and the seismic velocity response is equal to $\sim$ 19 days.

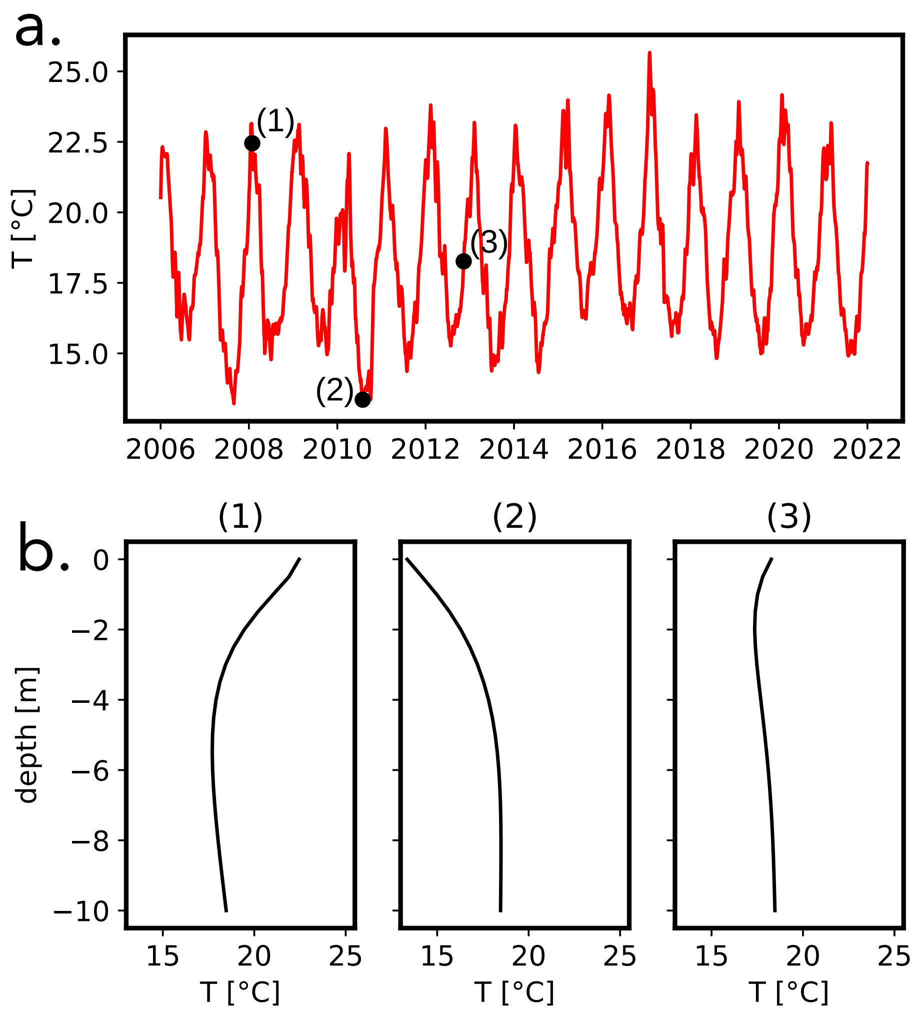

Figure S2. *Numerical simulation of the temperature diffusion under the seismic site.* **a.** shows the temperature time-series used in this study (taken at Iquique airport, $\sim$ 30 km from the PATCX station) to construct the dv/v models. The numbers indicate different dates for which the modelled temperature depth-profiles are shown in **b**.

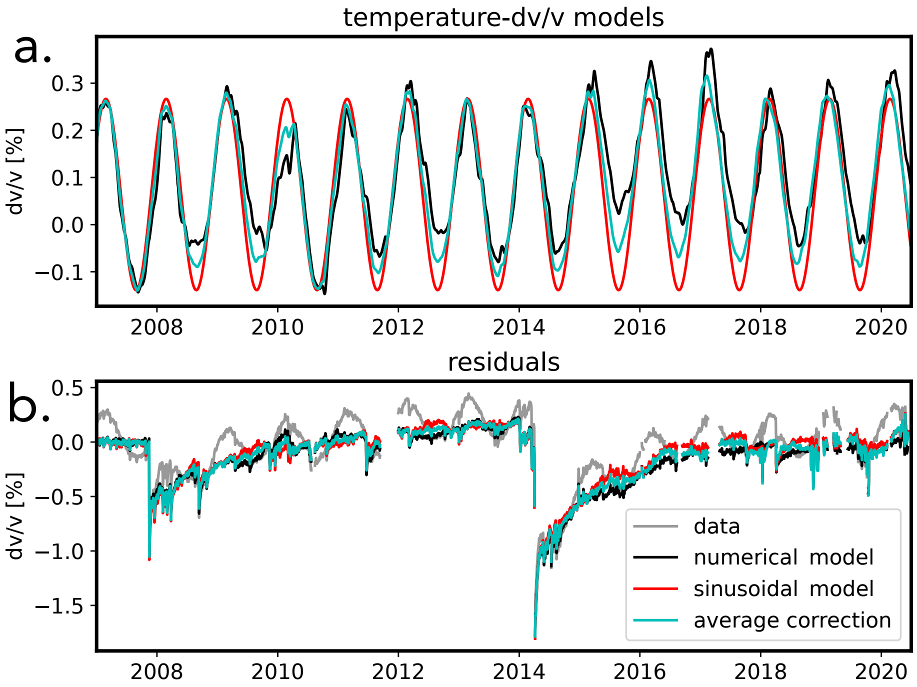

Figure S3. *Temperature-induced dv/v models* **a.** The solid lines show the different models for correcting the velocity changes due to surface temperature variations. The legend for the colours is indicated on the lower plot. **b.** The coloured lines represent the residuals of the data after correction for temperature effect sby the models shown on plot **a**. The raw data is shown in grey.

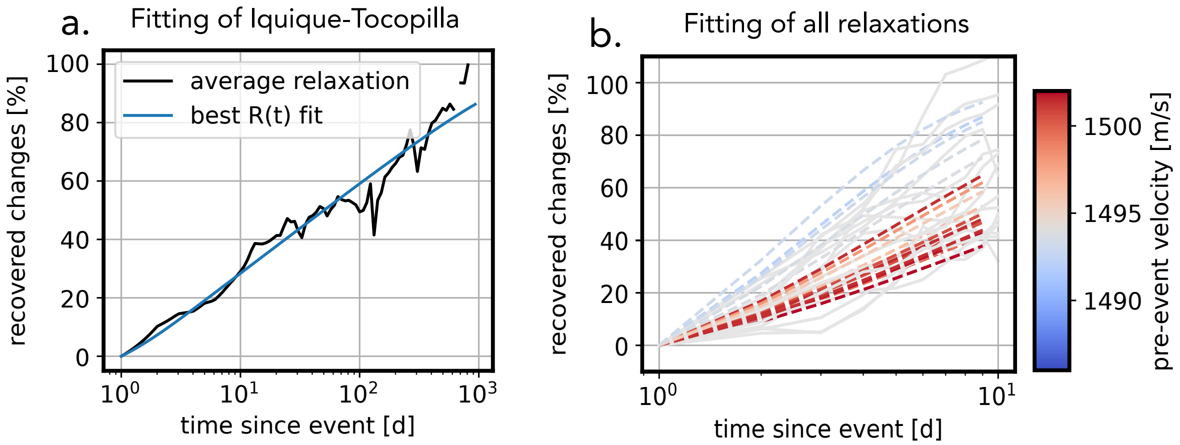

Figure S4. *Fitting of recoveries with the relaxation function R(t)* **a.** The black line shows the interpolated average velocity recovery observed after Tocopilla and Iquique earthquakes. The blue line shows the best $R(t)$ fit with $\tau_{\max} = 3887$ d. **b.** Fitting of all aftershocks-induced recoveries. The dashed lines indicate the fits. The pre-event velocity before the earthquakes are indicated in colours.

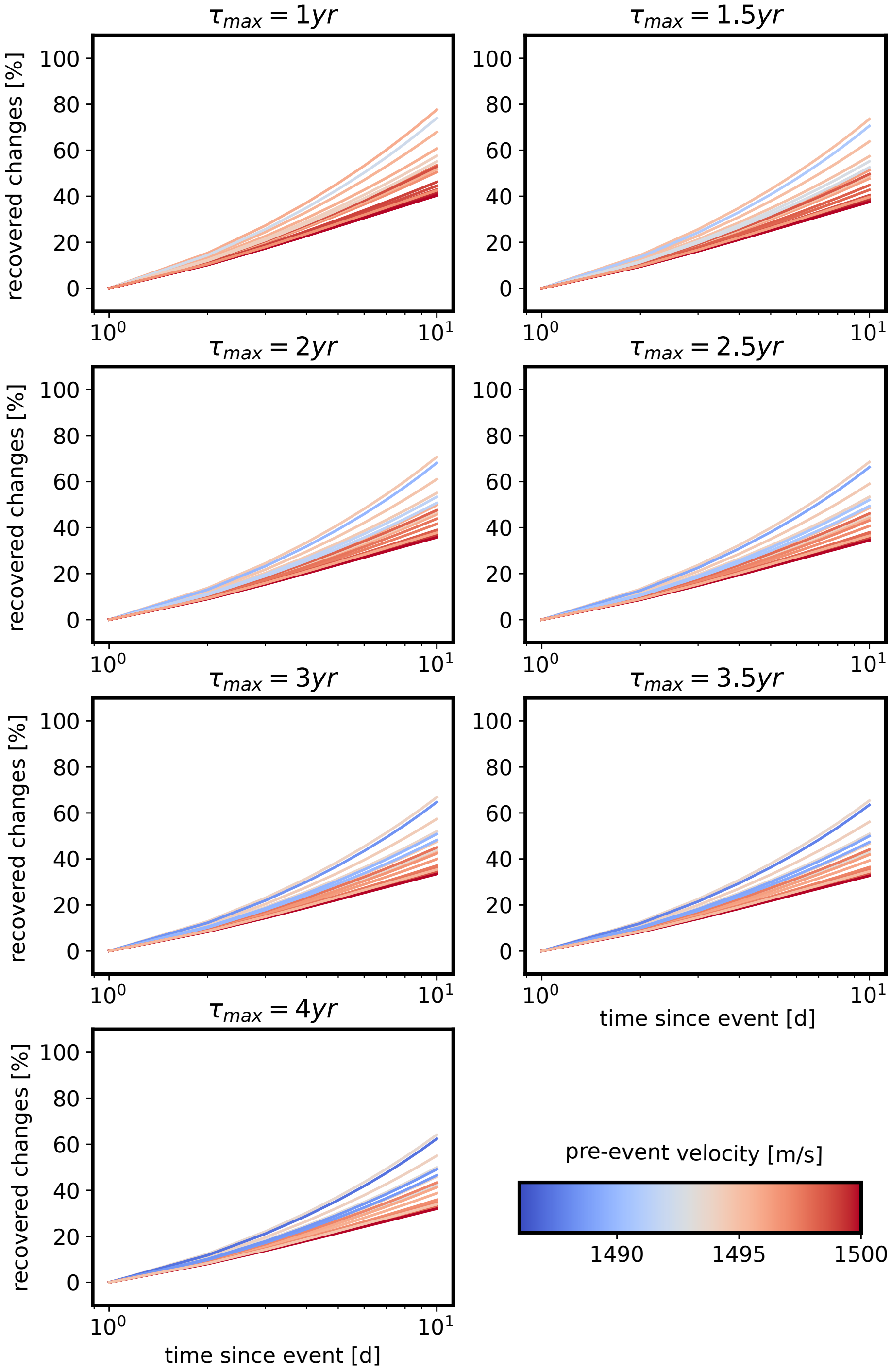

Figure S5. *Modelled recoveries after individual seismic events, normalised by the amplitude of the drop.* Each subplot corresponds to a different model characterised by a constant timescale of relaxation as shown in Figure 2d of the main text. The pre-event velocity before the earthquakes are indicated in colours.

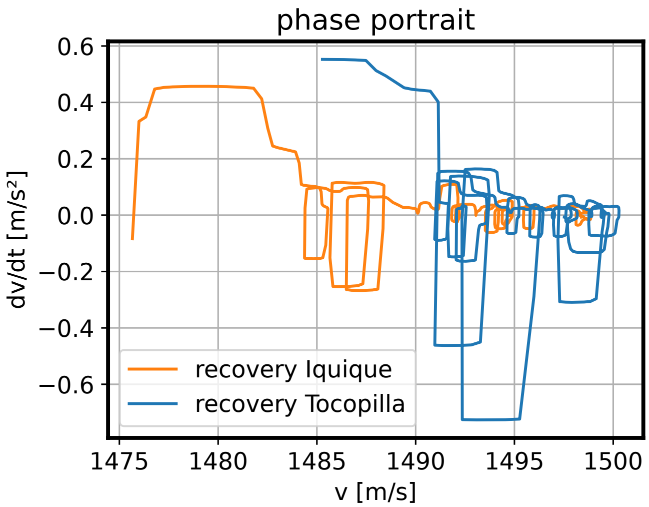

Figure S6. *Phase portrait (e.g dv/dt vs v plot) of the velocity changes observed after the Tocopilla and Iquique earthquakes.* Both lines were computed for the recoveries shown in Figure 1c of the main text. The derivative dv/dt was obtained numerically using the total-variation regularisation method[1].

Table S1. Aftershocks Information

date	mw	lat	lon
16.12.2007	6.7	-22.89	-70.13
04.02.2008	6.3	-20.19	-69.95
16.02.2008	6.1	-21.32	-68.36
01.03.2008	5.6	-20.31	-69.98
24.03.2008	6.2	-20.08	-68.97
10.09.2008	5.7	-20.3	-69.18
17.04.2009	6.1	-19.58	-70.5
15.07.2009	5.3	-20.38	-69.15
13.11.2009	6.9	-19.41	-70.3
22.10.2010	5.7	-20.83	-68.45
20.06.2011	6.5	-21.64	-68.35
10.03.2012	5.2	-19.67	-69.15
07.01.2014	5.3	-21.03	-69.61
23.03.2014	6.2	-19.7	-70.78
30.05.2014	5.6	-21.29	-70
13.07.2014	5.6	-20.26	-70.35
14.08.2014	5.3	-19.86	-69.86
10.10.2017	6.3	-18.52	-69.64
05.04.2018	5.6	-20.38	-70.61

References

1. Chartrand, R. Numerical differentiation of noisy, nonsmooth, multidimensional data. In *2017 IEEE Global Conference on Signal and Information Processing (GlobalSIP)*, 244–248, DOI: 10.1109/GlobalSIP.2017.8308641 (2017).
